# Magnitude and trend of perinatal mortality and its relationship with inter-pregnancy interval in Ethiopia: a systematic review and meta-analysis

**DOI:** 10.1186/s12884-020-03089-2

**Published:** 2020-07-29

**Authors:** Belayneh Hamdela Jena, Gashaw Andargie Biks, Kassahun Alemu Gelaye, Yigzaw Kebede Gete

**Affiliations:** 1 Department of Public Health, College of Medicine and Health Sciences, Wachemo University, Hossana, Ethiopia; 2 Department of Health System and Policy, Institute of Public Health, College of Medicine and Health Sciences, University of Gondar, Gondar, Ethiopia; 3 Department of Epidemiology and Biostatistics, Institute of Public Health, College of Medicine and Health Sciences, University of Gondar, Gondar, Ethiopia

**Keywords:** Inter-pregnancy interval, Perinatal mortality, Stillbirth, Early neonatal death, Ethiopia

## Abstract

**Background:**

Perinatal mortality remains a problem in Ethiopia. Findings of primary studies varied on level of perinatal mortality and its predictors including inter-pregnancy interval. The aim of this review was to estimate the pooled perinatal mortality rate, its trend overtime and verify the association with inter-pregnancy interval in Ethiopian context.

**Methods:**

Studies were accessed through the electronic web-based search strategies from PubMed, ScienceDirect, Hinari for health via Research4Life, Google and Advanced Google search, and retrieving via relevant references using a combination of medical subject headings (MeSH terms) and key words related with inter-pregnancy interval. R version 3.4.3 software was used for the meta-analysis. A forest plot and I^2^ test were done to assess heterogeneity. Sensitivity analysis and subgroup analysis were done to deal with heterogeneity. A weighted inverse variance random-effects model was applied to estimate pooled effect sizes. A funnel plot and Egger’s regression test were done to check publication bias.

**Results:**

A total of 34 studies used to answer review questions (30 for perinatal mortality rate and its trend estimation from 1997 to 2019 and 8 for its relationship with inter-pregnancy interval). The pooled perinatal mortality rate was 51.3 per 1000 total births (95% CI: 40.8–62.8). The pooled stillbirth rate was 36.9 per 1000 births (95% CI: 27.3–47.8) and early neonatal mortality rate was 29.5 per 1000 live births (95% CI: 23.9–35.6). Increasing trend was seen in stillbirth rate (23.7 to 36.9 per 1000 births) while decreasing trend in early neonatal mortality rate (51 to 29.5 per 1000 live births). Slight reduction trend was observed in overall perinatal mortality rate (66 to 51.3 per 1000 births). An inter-pregnancy interval less than 15 months was found to be statistically significantly associated with perinatal mortality; pooled OR = 2.76 (95% CI: 2.1–3.62). Spacing pregnancy for at least 15 months was related with reducing perinatal mortality by 64% (95% CI: 52.38, 72.38%).

**Conclusions:**

In Ethiopia, perinatal mortality rate remains high. Insignificant reduction trend was observed in overall perinatal mortality rate. Counseling couples about the importance of spacing pregnancy and intensifying long-acting contraceptive use will help in reducing perinatal mortality related to poor pregnancy spacing.

## Background

Perinatal mortality is defined as fetal loss at or after 28 weeks of gestation (stillbirth) or neonatal death within 7 days of life (early neonatal mortality) [1].

Globally, of the estimated 3 million perinatal deaths that occur in each year, low and middle income countries share the highest burden (97–99%). Perinatal mortality is a reflection of poor socio-economic status of a country, poor maternal health service utilization and the quality of obstetric and neonatal care facilities available [2–4]. Furthermore, inappropriate maternal health care provision during the course of pregnancy, labor, delivery and postpartum periods, particularly when complications happen and lack of newborn care immediately after delivery and within the first 7 days of life were the main contributing factors for the highest burden of perinatal death in low resource settings [4].

In Ethiopia, perinatal mortality is one of the highest in Africa, 46 per 1000 pregnancies [2]. It is mainly attributed to home delivery, which accounts for more than 75% of the perinatal deaths [5]. Reducing neonatal, infant and under five mortalities were a global agenda. Ethiopia shared sustainable development goal (SDG) to achieve the target for reduction of neonatal mortality to below 12 per 1000 live births, by 2030 [6]. Reduction of neonatal, infant and under-five mortalities may not be realized without substantial reduction of perinatal mortality. This is because of the fact that most of the neonatal deaths occur during the first week of life, which is a part of perinatal mortality [4, 7, 8]. Thus, all interventions aimed at reducing neonatal, infant and under-five mortalities are expected to begin with intervention during the perinatal periods. Of course, the Ministry Of Health, Ethiopia, had been working for years to make health services accessible for women through community and facility based interventions to increase newborn and child survival (Unpublished). Despite these interventions, perinatal mortality remains one of the problems in Ethiopia, particularly; home delivery remains the challenge to reduce perinatal mortality. Still, most (74%) women give birth outside health institution without skilled care attendant [9]. Studies indicated that the causes of stillbirth and early neonatal death are closely related [10] so that the risk factors for both of them can be studied together as perinatal mortality. With regard to this, studies identified multiple risk factors for perinatal mortality such as prematurity, low birth weight, previous history of perinatal death, not receiving tetanus toxoid immunization, lack of Iron supplementation etc. [1, 3, 11, 12]. On the other side, closely spaced pregnancies are hypothesized as one of the risk factors for poor perinatal outcomes [13]. Few studies conducted in Ethiopia attempted to see the relationship between inter-pregnancy interval and perinatal mortality. However, there were inconsistencies among these studies. Some studies reported that there was relationship [1, 3] while others reported no relationship [11, 12, 14]. To come up with evidence based interventions, clarifying the relationship is vital. In this regard, the researchers’ hypothesis was that; short inter-pregnancy interval of less than 15 months would have increased the risk of perinatal mortality as compared to inter-pregnancy interval of greater than or equal to 15 months in Ethiopia.

It is obvious that systematic review and meta-analysis yield higher level of evidence for policy and decision making. Evidence on the pooled effect sizes for overall perinatal mortality, stillbirth and early neonatal mortality was limited in Ethiopia. Cumulative evidence (trend) of perinatal mortality, stillbirth and early neonatal mortality were inadequate to see the progresses over time and give subsequent attention. More than half of Ethiopian women have the short interval between pregnancies [2]. Further attention is needed to increase inter-pregnancy interval. Thus, ascertaining its relationship with perinatal mortality is crucial.

Therefore, this systematic review and meta-analysis was aimed to address the following review questions:

1. What is the pooled estimate of a perinatal mortality rate in Ethiopia?
2. What was the trend of perinatal mortality over time in Ethiopia?
3. Was inter-pregnancy interval less than 15 months associated with perinatal mortality as compared to its counterpart in Ethiopia?

## Methods

### Reporting and protocol registration

The results of this systematic review and meta-analysis were reported based on the Preferred Reporting Items for Systematic Review and Meta-Analysis statement (PRISMA 2009) guideline with 27 items checklist [15] (See Additional file 1). The protocol was registered in the PROSPERO Database: (PROSPERO 2019: CRD42019125186) Available from: https://www.crd.york.ac.uk/PROSPERO.

### Inclusion criteria

Observational study designs (cross-sectional, case referent and cohort/follow up studies) were included in the review. Studies that observed the proportion of at least one of the perinatal outcomes (stillbirth and/or early neonatal death) and/or the relationship between short inter-pregnancy interval or birth interval and any one of the perinatal outcomes were considered. We considered articles published in English language, have relevant full text and from Ethiopia. There was no time restriction for the articles published in order to see the trend over time.

### Exclusion criteria

Studies that considered only high risk mother for perinatal death such as studies done on mothers with pregnancy related hypertensive disorders, ante-partum hemorrhage, obstructed labor etc. were excluded as these conditions have known effect on perinatal outcomes. Hence, it might exaggerate the effect size and the pooled estimates might be misleading. Qualitative studies were also excluded since it is not appropriate to quantify effect sizes. Finally, we did not consider interventional studies in this review.

### Information sources and search strategies

Databases (PubMed, ScienceDirect, and Hinari for health via Research4Life) and grey literatures sources (Google, Advanced Google Scholar) were accessed. Further search was made through snowballing or retrieving from relevant references used in related studies. A combination of medical subject headings (MeSH terms) using Boolean operators and key words related with inter-pregnancy interval were used to search studies.

The last date to access databases was made in December, 3/2019 on Tuesday until 2:50 PM. [See Additional file 2].

### Study selection

Retrieved studies were exported to reference manager software, Endnote version 7. Duplicated studies were removed using the Endnote and manually. Two independent reviewers (Belayneh Hamdela (BH) and Alemu Ersido (AE)) screened the title and abstract for the relevance. During this preliminary assessment, reading title and abstract, primary studies found to be irrelevant were excluded. Two reviewers (BH and AE) participated in study selection by reading titles and abstracts only. When disagreement between two reviewers happened, the third reviewer (Solomon Hailemeskel (SH)) was used to handle the disagreement based on the relevance for pre-specified objectives and inclusion criteria. Then primary studies with only relevant information and fulfills inclusion criteria were selected for full text review and reason for exclusion were presented using PRISMA flow diagram.

### Risk of Bias assessment

Two independent reviewers (BH and AE) appraised the quality of each study. The Joanna Briggs Institute (JBI) quality appraisal checklist was used for each study design [16]. The disagreement between two reviewers (BH and AE) was resolved by involving third reviewer (SH). Cohen’s Kappa statistics was used to calculate degree of agreement between the two reviewers (BH and AE).

For each of the three study designs, a study was considered low risk of bias score of 50% and above of the quality assessment indicators. Studies with low risk of bias were included for the meta-analysis.

### Data extraction process

Data extraction form was prepared and piloted using excel spread sheet. Two forms were prepared: one for the magnitude and trend of perinatal mortality (including stillbirth and early neonatal mortality), and the other form was for the risk factor of perinatal mortality (i.e. inter-pregnancy interval). The form prepared for perinatal mortality contained: study id, region, study setting, authors and year of publication, study design, sample size and proportion of perinatal mortality. As most women did not remember last normal menstrual period, inter-pregnancy interval (IPI) was usually estimated from the birth interval by subtracting 9 months of gestation [2]. In this review, the main exposure variable was inter-pregnancy interval less than 15 months or birth interval less than 24 months. With this regard, the other form, in addition to those mentioned above, contains data for 2 × 2 table: perinatal death among exposed (IPI < 15 months), survived among exposed, perinatal death among unexposed (IPI > = 15 months), survived among unexposed, exposed total, unexposed total and the Odds Ratios (ORs). Two reviewers (BH and AE) extracted the data on the forms and cross checked for any disagreements. The extracted data then edited and saved in a comma delimited (CSV) file format to suit for the analysis.

### Data synthesis and analysis

The data extracted and saved in CSV format in excel spread sheet was imported to R version 3.4.3 statistical software for the analysis. Before pooling the effect sizes, the summary measures were transformed to suitable transformations that result in normal distribution. Accordingly, the proportion of perinatal mortality was transformed to Freeman and Tukey double arcsine transformation (PFT). Double arcsine transformation was recommended for proportions when the observed proportions of each study are either below 0.20 or above 0.80. In such a cases log transformation might have a limitation of stabilizing variance. Therefore, Freeman and Tukey double arcsine transformation was recommended to solve the limitation of log transformation or to stabilize the variance so that it gives more valid estimate of the weighted average. On the other hand, the odds ratios of the individual studies were transformed in to its log scale. All analyses were done by using transformed summary measures. Back translation of summary measures was done for reporting as this help readers easily understand the results. Heterogeneity among studies was checked by two methods. Firstly, subjective method (forest plot) was used to visualize graphically. Secondly, more objectively tested and quantified using the I-squared statistic, in which 25, 50, and 75% represented low, moderate and high heterogeneity respectively [17]. Random effects model was used to estimate the pooled effect size using Dar-Simonial Liard (DL). Tau squared was used to quantify the amount of heterogeneity in random effect model. To deal with heterogeneity, sensitivity and subgroup analyses were done. Sub group analysis was done by using year of publication, study settings, regions and designs. To report the pooled effect sizes from the included studies, meta-analysis of pooled proportion and odds ratio were done. Potential publication bias was checked using funnel plot and Egger’s regression test. Overall trends of perinatal mortality rates were examined both graphically and using simple linear regression test. Data for trend analysis is available from 1997 to 2019 years. Since there was no data for all years we grouped cumulative trends for every 5 years to suit for better interpretation of trend line. *P*-value less than 0.05 was used to describe statistically significant decreasing trend in perinatal mortality rate.

## Results

### Selection of studies

A total of 918 studies were retrieved from sources by pre-specified search strategies. Of these, 47 studies were duplicates and are removed. In preliminary screening, 815 studies (802 by looking at the title and 13 by abstract) were removed. A total of 56 studies were identified for checking eligibility for full text review. Of these, 21 studies were excluded with the following reasons:

A case control study from Bonga General and Mizan Tepi University Teaching Hospitals, a facility based case control study from Arbaminch general hospital, a prospective cohort study from tertiary care hospitals in Addis Ababa, a case control study from public hospitals of Tigray, a prospective cohort study from Northern Ethiopia, a cross-sectional study on neonatal mortality from Ethiopia, a case control study from Debre Tabor town, a hospital based retrospective cohort study from Somali Ethiopia, a case control study from Hawassa University hospital were excluded due to lack of relevant data for this review. A 5 years retrospective survey from Tikur Anbesa Hospital, a matched case control study from Kalu district, a retrospective chart review from Yekatit 12 hospital, Addis Ababa, a cross-sectional study from Addis Ababa hospital, a cross-sectional study from Jimma Hospital and a retrospective review on perinatal mortality audit from Jimma hospital were excluded due to lack of access to full text. A retrospective study from Mettu Karl referral hospital Ethiopia, a cross-sectional study from Amhara region hospitals, an institutional based cross-sectional study from Debremarkos referral hospital and a retrospective cohort study from Wolita Sodo referral hospital were excluded since they were conducted on high risk groups for perinatal deaths. A study from Kersa demographic and health surveillance system site was excluded due to double reporting in publications. Lastly, an interventional study from Ethiopia was excluded because of the design as this review focus on observational studies.

**Fig. 1:**
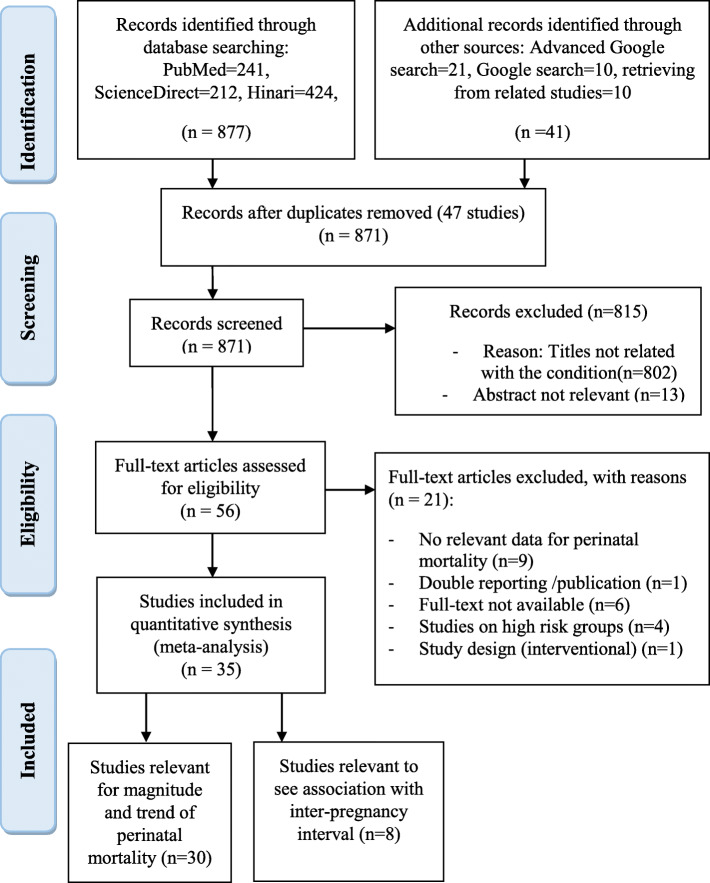
Study selection procedure for the systematic review and meta-analysis, Ethiopia

Finally, a total of 35 studies that fulfill the pre-specified inclusion criteria were obtained (Fig. 1).

### Characteristics of included studies

A total of 35 studies included in this review. However, studies included in the meta-analysis were thirty four [1–3, 8, 11, 12, 18–45]. Pregnant women, women attending for labor and delivery service and newborns were study participants considered for data source in primary studies.

Of 34 studies included in the meta-analysis, 30 studies contained data relevant for perinatal mortality and trend estimation. From 30 studies, 25(83.3%) and 18(60%) studies had data relevant for pooling stillbirth and early neonatal mortality rates, respectively. One study (25) had crudely reported perinatal mortality without specifying either stillbirth or early neonatal mortality. From 34 studies, only 8(23.5%) contained data relevant to see the relationship between inter-pregnancy interval and perinatal mortality.

Perinatal mortality rate (PMR) in primary studies ranges from 13.98 (15) to 85.3 (24) per 1000 total births in community based studies. Whilst it ranges from 27.6 (25) to 173 (23) per 1000 total births in health institution based studies. Stillbirth rate (SBR) ranges from 5.9 (26) to 85.3 (24) per 1000 total births in community based studies while from 22.7 (31) to 173.3(23) per 1000 total births at health institution based studies. Early neonatal mortality rate (ENMR) ranges from 10.8(26) to 41.4(32) per 1000 live births in community based studies while it ranges from 24.8(5) to 110(30) per 1000 live births at health institution. All conditions (PMR, SBR and ENMR) were higher in health facility based studies than community based studies. Inter-pregnancy interval less than 15 months or birth interval less than 24 months was significantly associated with perinatal mortality in 5(62.5%) of the 8 studies. In two studies however (20, 26) it was not significantly associated with perinatal mortality. Categorization of inter-pregnancy interval or birth interval in these two studies was not in line with most of the literatures. In one study (34), it is unclear that, whether it was fitted in the adjusted model or not.

Of 34 studies, 26(76.4%) were conducted in both (urban and rural) settings. The others: 4(11.8%) in urban and 4(11.8%) in rural settings alone. Sixteen (47.1%) were community based while 18(52.9%) were institution based studies. Regarding regions: Amhara 8(23.6%), Oromia 6(17.7%), SNNP 5(14.7%), Addis Ababa 3(8.8%), Tigray 5(14.7%), Benishangul-Gumuz 1(2.9%), mixed (more than one region) 3(8.8%) and the others 3(8.8%) were National (Ethiopia) Demographic and Health Surveys (EDHS). Concerning the study design: cross-sectional 21(61.8%), follow ups 6(17.6%), prospective cohort 1(2.9%), case control 4(11.8%), nested case control 2(5.9%).

Regarding year of publication: 2(5.9%) published before 2000, 11(32.3%) published from 2001 to 2015 and 21(61.8%) published from 2016 to 2019.

Study id, author, year of publication, region, study design, sample size, exposure variable and outcome data were extracted to describe the characteristics of the studies. The total sample size of the studies was 141,835 (136,168 to determine the magnitude of perinatal mortality and 5667 to see the relationship between inter-pregnancy interval and perinatal mortality). A sample size considered for primary studies ranges from 300 to 20,161. For the assessment of the relationship between inter-pregnancy interval and perinatal mortality, the sample size considered ranges from, 219 to 1949 (See Table 1).

**Table 1:** Characteristics of primary studies included in the systematic review and meta-analysis, 2019, Ethiopia

Study ID	Author (year)	Region	Study Design	Sample Size	PMR per 1000 total births	SBR per 1000 births	ENM R per 1000 live births	IPI < 15 months with PM	IPI < 15 months without PM	IPI ≥ 15 months with PM	IPI ≥ 15 months without PM	OR [95% CI]
1	Sahlemariam Y (1997) [36]	Addis Ababa	Follow up	1606	106/1606 **(66)**	38/1606 **(23.7)**	68/1334 **(50.9)**	–	–	–	–	–
2	EDHS (2000) [32]	National	Cross sectional	12,494	655/12494 **(52)**	234/12494 **(18.7)**	421/12260 **(34)**	–	–	–	–	–
3	EDHS (2005) [33]	National	Cross sectional	11,280	420/11280 **(37)**	117/11280 **(10.4)**	303/10860 **(27.9)**	–	–	–	–	–
4	EDHS (2011) [2]	National	Cross sectional	12,077	551/12077 **(46)**	204/12077 **(16.9)**	347/11873 **(29)**	–	–	–	–	–
5	Chekol A (2011) [26]	Benishangul	Cross sectional	581	69/581 **(118.8)**	56/581 **(96.4)**	13/525 **(24.8)**	–	–	–	–	–
6	Assefa N (2012) [22]	Oromia	prospective follow up	1438	27/1438 **(18.8)**	27/1438 **(18.8)**	–	–	–	–	–	–
7	Wakgari N (2013) [41]	mixed (EDHS)	Cross sectional	8651	343/8651 **(39.6)**	–	343/8651 **(39.6)**	–	–	–	–	–
8	Andargie G (2013) [3]	Amhara	Prospective longitudinal	1752	88/1752 **(50.2)**	41/1752 **(23.4)**	47/1711 **(27.5)**	40	483	48	1881	2.04 [1.32; 3.14]
9	Worku A (2014) [44]	Amhara	Prospective Cohort	727	36/727 **(49.52)**	20/727 **(27.5)**	16/707 **(22.6)**	–	–	–	–	–
10	Adane A (2014) [19]	Amhara	Cross sectional	481	34/481 **(70.7)**	34/481 **(70.7)**	–	–	–	–	–	–
11	Yaya Y (2014) [45]	SNNP	Cross sectional	11,762	226/11762 **(19.2)**	226/11762 **(19.2)**	–	–	–	–	–	–
12	Debelew G (2014) [28]	Oromia	Prospective follow up	3510	123/3510 **(35)**	47/3510 **(13.4)**	76/3463 **(21.9)**	–	–	–	–	–
13	Abdo R (2016) [18]	SNNP	Cross sectional	327	28/327 **(85.6)**	28/327 **(85.6)**	–	–	–	–	–	–
14	Mengesha H (2016) [34]	Tigray	prospective cohort	1152	50/1152 **(43.4)**	–	50/1152 **(43.4)**	–	–	–	–	–
15	Shifa G (2016) [8]	SNNP	Cross sectional	20,161	282/20161 **(13.98)**	–	282/20161 **(13.98)**	–	–	–	–	–
16	Berhie K (2016) [25]	mixed	Cross sectional	12,560	320/12560 **(25.5)**	–	320/12560 **(25.5)**	–	–	–	–	–
17	Asefa D (2016) [21]	Oromia	Cross sectional	9921	1080/9921 **(108.9)**	852/9921 **(85.9)**	232/9069 **(25.6)**	–	–	–	–	–
18	Aragaw Y (2016) [20]	Oromia	Cross sectional	3786	372/3786 **(98.3)**	268/3786 **(70.8)**	104/3518 **(29.6)**	–	–	–	–	–
19	Ballard K (2016) [23]	mixed (Amhara and Oromia)	Cross sectional	4358	95/4358 **(21.8)**	95/4358 **(21.8)**	–	–	–	–	–	–
20	Yirgu R (2016) [12]	Amhara	Nested Case control	4049	102/4049 **(25.2)**	57/4049 **(14.1)**	45/3992 **(11.3)**	1	5	101	217	0.43 [0.05; 3.73]
21	Cherie N (2017) [27]	Amhara	Cross sectional	462	21/462 **(45.5)**	21/462 **(45.5)**	–	–	–	–	–	–
22	Dejene T (2017) [38]	Oromia	Cross sectional	413	33/413 **(79.9)**	33/413 **(79.9)**	–	–	–	–	–	–
23	Mihiretu A (2017) [35]	SNNP	Cross sectional	300	52/300 **(173.3)**	52/300 **(173.3)**	–	–	–	–	–	–
24	Lakew D (2017) [31]	Amhara	Cross sectional	2555	218/2555 **(85.3)**	218/2555 **(85.3)**	–	104	793	43	1009	3.08 [2.13; 4.44]
25	Tsegaye B (2018) [40]	SNNP	Cross sectional	580	16/580 **(27.6)**	–	–	–	–	–	–	–
26	Roro E (2018) [11]	Oromia	Nested Case control	4383	73/4383 **(16.7)**	26/4383 **(5.9)**	47/4357 **(10.8)**	15	18	58	128	1.84 [0.87; 3.9]
27	Goba G (2018) [1]	Tigray	unmatched case control	265	–	–	–	42	39	49	135	2.97 [1.72; 5.12]
28	Getiye Y (2017) [29]	Addis Ababa	unmatched case control	395	–	–	–	41	29	69	256	5.25 [3.04; 9.04]
29	Tilahun S (2008) [39]	Addis Ababa	unmatched case control	343	–	–	–	27	27	93	196	2.11 [1.17; 3.79]
30	Tewabe T (2018) [37]	Amhara	Cross sectional	391	43/391 **(109.9)**		43/391 **(109.9)**	–	–	–	–	–
31	Haftu A (2018) [30]	Tigray	prospective cohort	1103	55/1103 **(49.9**)	25/1103 **(22.7)**	30/1078 **(27.8)**	–	–	–	–	–
32	Woldeamanuel B (2019) [42]	Mixed (EDHS)	Cross sectional	2738	170/2738 **(62.09)**	59/2738 **(21.55)**	111/2679 **(41.43)**	–	–	–	–	–
33	Berhe T (2019) [24]	Tigray	cross sectional	570	21/570 **(36.8)**	21/570 **(36.8)**		–	–	–	–	–
34	Worede D (2019) [43]	Amhara	unmatched case control	420				49	98	35	238	3.4 [2.08; 5.57]

National: Study conducted at national level (Ethiopia) like DHS

Mixed: when study considered data for more than one region in Ethiopia

### Quality (risk of bias) assessment for the included studies

Of 24 cross-sectional studies: except for 4 studies (ID: 2, 24, 15, 33), measurement of exposure in a valid and reliable way was unclear for 16 (66.6%) studies. The confounder factors identified way was unclear for 9 studies (ID: 21, 5, 22, 11, 17, 18, 23, 19, 16). Strategies to deal with confounding factors were also unclear for 2 studies (ID: 17, 19). None of the case control studies used matching for potential confounders. From the follow up studies; strategies to address the incomplete follow up was not utilized in 4 studies (ID: 1, 6, 9, 14). According to Cohen’s Kappa statistics (K = 0.65, % of agreement = 97.2%), the two reviewers (BH and AE) have “substantial agreement” to include studies in the meta-analysis [46]. One study [47] was not considered for data extraction since it scores below 50% for JBI quality indicators. See Table S1 in Additional file 3 for quality assessment scores.

### Pooled estimate of perinatal mortality rate

Forest plot was used to identify heterogeneity between studies. The result of the forest plot indicated that there is heterogeneity among studies (I^2^ = 99%, *p* < 0.0001). The result in fixed effect suggests the need to fit the random effect model to handle heterogeneity.

In random effect model, tau^2^ using Dar-Simonial Liard (DL) quantified the amount of total heterogeneity to be 0.0046. Using double arcsine transformed proportion in random effect model, the weighted average from each study using the inverse of variances yield a more valid effect size.

**Fig. 2:**
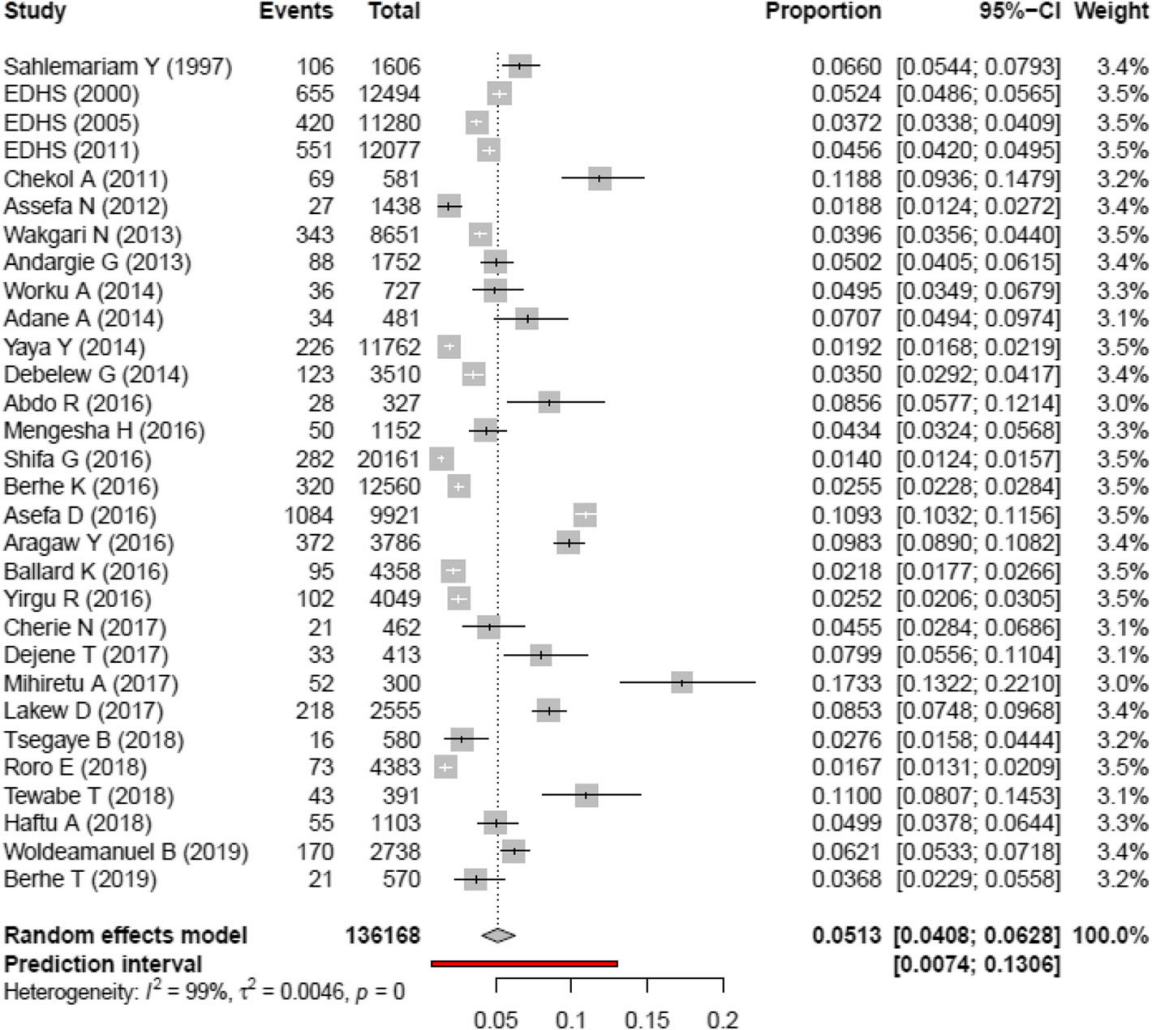
Forest plot showing heterogeneity among included studies to estimate perinatal mortality rate, 1997–2019, Ethiopia. In Fig. 2: Each squared box indicates the sample size of individual studies. The horizontal line at the middle of each box indicates the 95% confidence interval of individual studies. The dot at the middle of the squared box and the horizontal line indicates the effect size (PMR) of individual studies. The long horizontal line inside the squared box which is wider than the squared box indicates the sample size is smaller than expected. The diamond shape at the end of the broken vertical line indicates the pooled effect size (pooled PMR). The red color horizontal line shows the prediction interval. The boxes at the left side of the broken vertical line show the effect sizes of individual studies that are lower than the pooled effect size and vice versa

Accordingly, the pooled perinatal mortality rate in Ethiopia was 51.3 per 1000 total births with 95% CI (40.8, 62.8) (Fig. 2). Perinatal mortality includes stillbirth and early neonatal death. The pooled stillbirth rate was 36.9 per 1000 births with 95% CI (27.3, 47.8). The pooled early neonatal mortality rate was 29.5 per 1000 live births with 95% CI (23.9, 35.6).

### Sensitivity analysis

The result of sensitivity analysis indicated that no study is found to be removed since the estimate of each study, when removed, is within the confidence interval of the pooled perinatal mortality rate (40.8, 62.8) (Figure S1 in Additional file 4).

### Subgroup analysis

The results of subgroup analysis for magnitude of perinatal mortality rate was indicated below (Table 2).

**Table 2:** The pooled perinatal mortality rate for subgroup analysis in random effect model, Ethiopia

Variables	Characteristics	Pooled perinatal mortality rate per 1000 total births (95% CI)	Tau^**2**^
Year of publication	1997–2000	57.9 (45.4–71.9)	0.0003
	2001–2014	43.6 (33.9–54.4)	0.0015
	2015–2019	54.8 (36.9–76)	0.0083
Study place	Community	35 (27–44.1)	0.0022
	Institution	75 (58.6–93.2)	0.0035
Region	National DHS	44.9 (36.7–53.9)	0.0003
	Addis Ababa	66 (54.4–78.7)	Not applicable
	Amhara	59.1 (37.1–85.8)	0.0045
	Benishangual	118.8 (93.6–146.4)	Not applicable
	Oromia	53.2 (22–96.8)	0.0107
	SNNP	46.2 (28.6–67.6)	0.0024
	Tigray	49.1 (38.6–60.8)	0.0005
	Mixed^a^	28.6 (19.4–39.5)	0.0007
Study design	Cross-sectional	58 (44.2–73.6)	0.0051
	Follow up	43.4 (32.3–55.9)	0.0013
	Nested Case Control	20.7 (13.1–29.9)	0.0004
Study setting	Urban	45.5 (15.5–90)	0.0039
	Rural	20.5 (17.4–23.8)	0.0001
	Both	58.6 (45.7–72.8)	0.0049
Study outcome	Stillbirth	51.4 (36.2–29)	0.0035
	ENM	44.4 (21.6–74.8)	0.0041
	Both(PM)	52.7 (39.3–68)	0.0039

Mixed^a^: When the study considered more than one region

### Publication bias

The presence of small study effects/publication bias was examined using regression test (Egger test). The test result indicated that there was no any small study effects or publication bias (*P* = 0.076).

### Trend of perinatal mortality

**Fig. 3:**
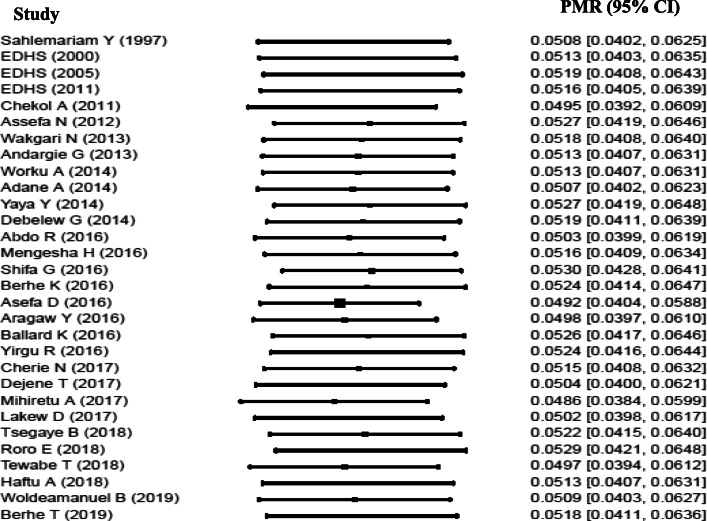
Trend of perinatal mortality rate, 1997-2019, Ethiopia. In fig. 3: The diamond shaped box and respective estimates across the junctions show the trend of perinatal mortality rate (PMR) per 1,000 total births for each corresponding year of publication.

**Fig. 4:**
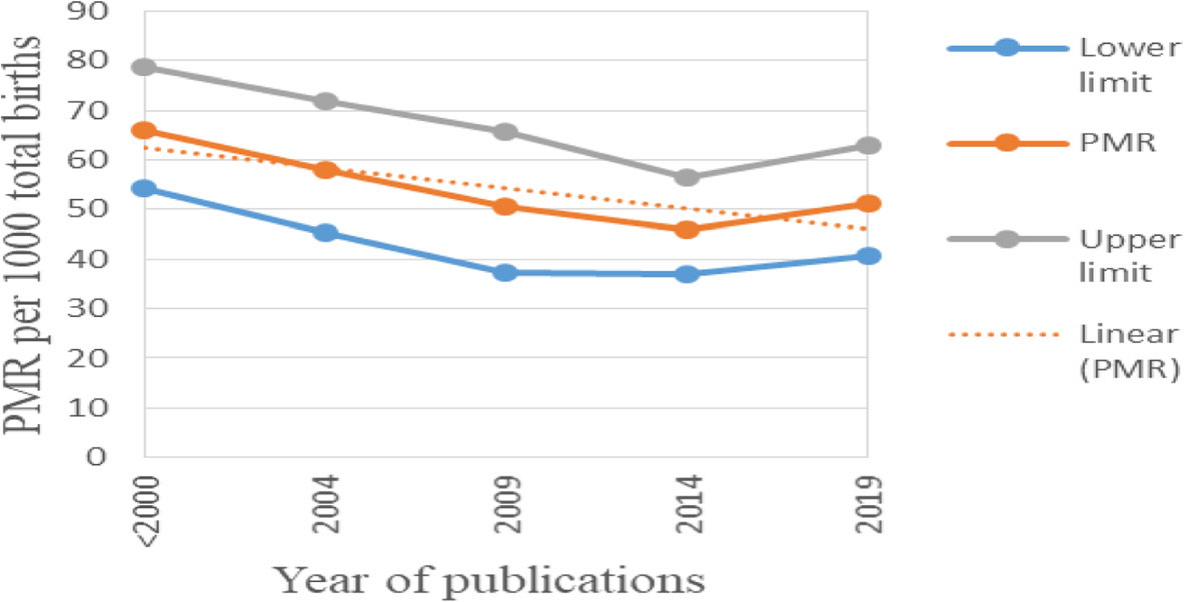
Trends of stillbirth rate (SBR) and early neonatal mortality rate (ENMR), 1997–2019, Ethiopia. In Fig. 4: The diamond shaped box and respective estimates across the junctions show the trend of stillbirth rate (SBR) per 1000 births and early neonatal mortality rate (ENMR) per 1000 live births for each corresponding year of publication

The cumulative meta-analysis indicated that, there is slight reduction trend with some ups and downs in between years of publication. From the Fig. 3, we can see that; perinatal mortality rate was reduced from 66 per 1000 total births in 1997 to 51.3 per 1000 total births in 2019. Significant reduction trend in perinatal mortality rate was observed from 1997 to 2014 (*p* = 0.014) but in the last 5 years (2015–2019) no reduction was seen. Overall, regression test indicated that there was no statistically significant reduction trend in perinatal mortality rate from 1997 to 2019 (*P* = 0.08). Since perinatal mortality rate was the sum of stillbirths and early neonatal mortality rates it is important to see the trends of them separately. In this regard, different kinds of trends were observed for stillbirth and early neonatal mortality rates (Fig. 4). There was no significant reduction trend in stillbirth rate rather it increased from 23.7 in 1997 to 36.9 in 2019 per 1000 births (*P* = 0.25). A significant reduction trend was observed in early neonatal mortality rate from 51 in 1997 to 29.5 in 2019 per 1000 live births (*P* = 0.01).

### Relationship between inter-pregnancy interval and perinatal mortality

**Fig. 5:**
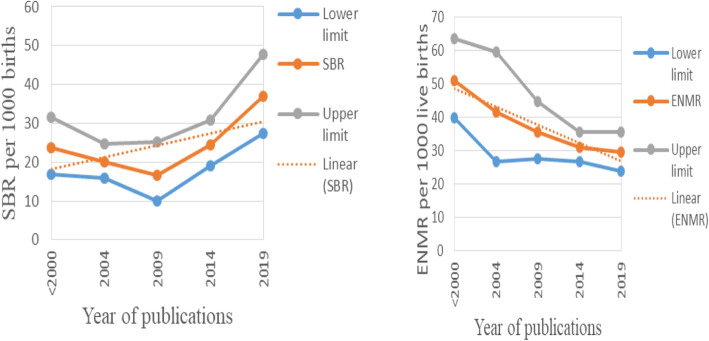
Forest plot showing heterogeneity for the relationship between inter-pregnancy interval and perinatal mortality, Ethiopia. In Fig. 5: Description of the figure is similar with that of Fig. 2 with the following exceptions: the full vertical line indicates no association. A horizontal line of squared box crossing the full vertical line indicates 95% CI crossing 1 (i.e. no association)

The relationship between inter-pregnancy interval less than 15 months and perinatal mortality was observed. Accordingly, children who were conceived within 15 months of their preceding child birth were nearly three times more likely risked to death during perinatal periods as compared to their counter parts; OR = 2.76, 95% CI: (2.1, 3.6). There is low heterogeneity among studies (I^2^ = 46%, *P* = 0.07) (Fig. 5).

### Publication bias

**Fig. 6:**
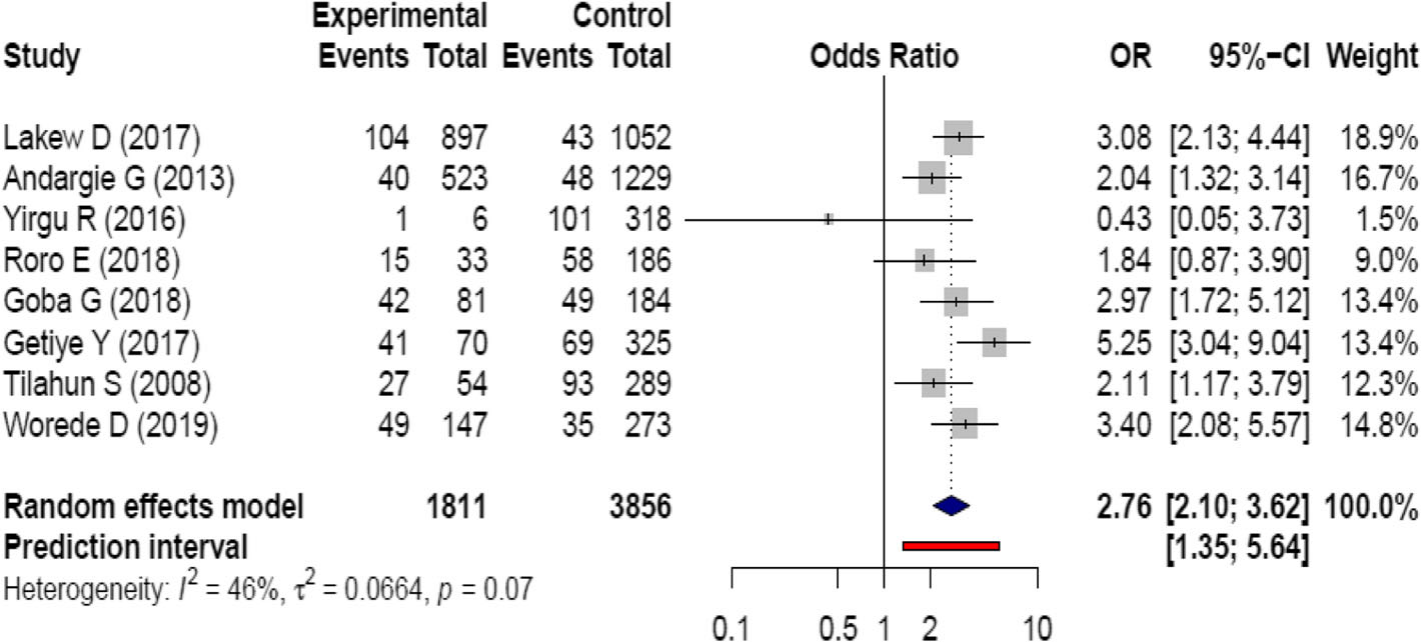
Funnel plot showing publication bias for the relationship between inter-pregnancy interval and perinatal mortality, Ethiopia. In Fig. 6: Each black dot represents a single study. The y-axis is the standard error of the effect estimate. The x-axis is transformed effect size (log odds ratio). Larger studies (large sample size) with higher power are placed towards the top and lower studies (small sample size) with lower power are placed at the bottom. Symmetrical distribution of dots on the both sides of the vertical line inside the triangle (funnel) shows relatively no publication bias (subjective)

Regression test for funnel plot asymmetry showed that there is no evidence of publications bias for trim and fill analysis (*P* = 0.313) (Fig. 6).

### Key findings of the review

Based on the review questions the following findings were obtained (Table 3).

**Table 3:** Key findings of the review

Variables	Effect sizes	95% CI
Perinatal mortality rate	51.3 per 1000 total births	40.8, 62.8
Stillbirth rate	36.9 per 1000 births	27.3, 47.8
Early neonatal mortality rate	29.5 per live births	23.9, 35.6

Women who have had pregnancy within 15 months of their preceding child birth were 2.76 times more likely faced perinatal mortality (stillbirths and early neonatal deaths) as compared to their counterparts (OR 2.76, 95%CI: 2.1, 3.6)

## Discussion

In this review, the pooled estimate of perinatal mortality rate, stillbirth rate and early neonatal mortality rate remained high. An increasing trend was seen in stillbirth rate while decreasing trend in early neonatal mortality rate from 1997 to 2019. Insignificant reduction trend was observed in overall perinatal mortality rate. An inter-pregnancy interval of less than 15 months was found to be statistically significantly associated with perinatal mortality.

Accordingly, the pooled perinatal mortality rate in Ethiopia was 51.3 per 1000 total births (stillbirths and live births). The result was higher than the reported pooled estimate for 21 countries in four sub-regions of Sub-Saharan Africa (SSA), 34.7 per 1000 births [48]. It is also higher than the reported pooled estimates for East African (34.5), West Africa (35.7), South Africa (30.3) and Central Africa (30.7) per 1000 births [48]. This implies that Ethiopia is a country with the highest burden of perinatal mortality in the African region. The differences might be due to the nature of studies considered for the meta-analysis. For this review, we considered published articles in addition to EDHS report. But the review in SSA considered only DHS reports for each country. The sample size and the settings considered across the regions might be the possible reasons for the difference in pooled estimates. In addition to that differences in awareness of the community, accessibility and utilization of maternal and child health services across the regions might be varied. In a country with large number of population, like Ethiopia, access to and quality of health services could be difficult. The topography and geographic location of health facilities together with lack of transportation in cases of emergencies might contribute for high perinatal death in Ethiopia. Home delivery is one of the main reasons for high prevalence of perinatal mortality because most of the complications that result in perinatal death happen during labor and delivery. Delay in seeking health facility delivery could also be the reason as most women in Ethiopia seek health care in cases of labor complication. Newborns couldn’t get the medical care that they need during emergencies when they born at home and are likely to die. Even if most women visit antenatal care, they did not give birth at health facilities. Most of the time women give birth at health facilities when they had bad pregnancy outcome or complications during their previous child birth. Otherwise, home delivery is considered as a norm because when women give births at home they get various kind of social support from significant others. This social support is lacked when they visit health facility.

The trend of a perinatal mortality rate in Ethiopia indicated that there was no sharp decline rather some ups and downs seen during different times. Studies from surveillance sites in Ethiopia showed that no more changes over time regarding perinatal mortality, especially stillbirth [5]. In this review, it is also observed that the trend of stillbirth remains alarmingly increasing. However, reduction trend was observed in early neonatal mortality. Therefore, up and downs trend in perinatal mortality rate was probably due to different patterns of trends in stillbirth and early neonatal mortality rates. Decreasing trend in early neonatal mortality rate might be the reflection of due emphasis given for the reduction of neonatal mortality in order to achieve MDGs and SDGs. A specifically targeted goal was lacking for the reduction of stillbirth. Thus, stillbirth needs due emphasis of policy actions. The subgroup analysis indicated that perinatal mortality at the community and health facility levels were 35 and 75 per 1000 total births respectively. This review showed some reduction as compared to the previous systematic review done in Ethiopia that indicated no reduction in perinatal mortality over decades rather it was stagnated at 40 and 90 per 1000 total births at community and health facility respectively [49]. The difference might be due to the difference in overall sample size, inclusion criteria, time considered to include primary studies, data sources accessed for the reviews and the effect of health interventions. In overall, consistent reduction is lacking in perinatal mortality, particularly stillbirth.

In this review, we observed that, children who were conceived within 15 months of their preceding child birth were nearly three times more likely risked to death during the perinatal period as compared to their counter parts [OR = 2.76, 95% CI: 2.1–3.6]. This result was supported by study conducted in Tanzania [13]. However, all primary studies included considered short inter-pregnancy interval less than 15 months with inter-pregnancy interval greater than or equal to 15 months. This categorization lacked international comparison since inter-pregnancy interval has not yet standardized. Other intervals also need to be studied to identify the optimal interval.

The National policy and decision makers need to understand an observed perinatal mortality rate in Ethiopia is one of the highest in Africa. Unless perinatal mortality is reduced, reducing neonatal mortality might be difficult since most of the neonatal mortality occurs within the first week of life that is a part of perinatal death. Existing interventions also need to be evaluated since there was no significant reduction in perinatal mortality (mainly stillbirth) for decades. All future interventions that are aimed at improving maternal and child health status need to consider perinatal health. Due emphasis also needed to be given for modern contraceptive utilization so as to enhance perinatal health because modern contraception is the main tool that we have at hand to reduce perinatal mortality related with poorly spaced pregnancies.

This review revealed that spacing pregnancies for at least 15 months was associated with reducing perinatal mortality by 64% (95% CI: 52.38, 72.38%). Further achievement can be obtained by increasing pregnancy interval for up to 24 months as recommended by World Health Organization. In the absence of randomized controlled trial that provide strong evidence, observational studies provide the valid estimate [50]. Therefore, this review could provide moderate evidence on the problem studied with Ethiopian context.

This meta-analysis may have some limitations since it is limited to observational studies published and some local sources might not be accessed. Estimated magnitude of perinatal mortality in Ethiopia may be lacked national representativeness because no data were found for regions not included in the final analysis. Time-trend analysis might not reflect the exact trend because all the years did not have reported data. Lack of controlling for confounding factors might affect the pooled effect size for the relationship between inter-pregnancy interval and perinatal mortality. Estimating inter-pregnancy interval from birth interval might result in some biases as perinatal deaths are commonly occur among preterm deliveries. Interpretations of the estimates need to consider contextual limitations. Beyond its limitation, this review will provide useful information for policy and decision makers, local planners and health workers to give due attention for prevention.

## Conclusions

In this review, a perinatal mortality rate remains high in Ethiopia. Insignificant reduction trend was observed in overall perinatal mortality rate from 1997 to 2019. Stillbirth rate was alarmingly increasing while early neonatal mortality rate was promisingly decreasing. Spacing pregnancy for at least 15 months was associated with reducing perinatal mortality by 64%. Counseling women on importance of spacing and strengthening modern contraceptive use will help in reducing perinatal mortality related to poor pregnancy spacing.

## Supplementary information

**Additional file 1:** Preferred Reporting Items for Systematic Review and Meta-Analysis statement (PRISMA 2009) guideline with 27 items checklist.

**Additional file 2:** Advanced search string.

**Additional file 3: Table S1.** Weaknesses of primary studies and JBI quality scores.

**Additional file 4: Figure S1.** Sensitivity Analysis.

## Data Availability

The datasets prepared and analyzed for this review are available in Table 1 and also from the corresponding author up on request reasonably.

## Abbreviations

EDHS: Ethiopia Demographic and Health Survey
ENMR: Early neonatal mortality rate
IPI: Inter-pregnancy interval
MeSH: Medical Subjects Heading
PMR: Perinatal mortality rate
SBR: Stillbirth rate
