# Supplementary material for: Magnitude and trend of perinatal mortality and its relationship with inter-pregnancy interval in Ethiopia: a systematic review and meta-analysis
Source: BMC Pregnancy Childbirth. 2020 Jul 29;20:432. doi: 10.1186/s12884-020-03089-2 (PMC7389567; doi:10.1186/s12884-020-03089-2)
Supplement: Supplementary file 2 — Additional file 2: Advanced search string. [file 12884_2020_3089_MOESM2_ESM.docx]

**Additional file 2. Advanced search string**

Advanced search strategies using Boolean operators in PubMed: ((((((((((((((((((((((interpregnancy interval*[MeSH Terms]) OR inter-pregnancy interval*[MeSH Terms]) OR pregnancy interval*[MeSH Terms]) OR pregnancy spacing*[MeSH Terms]) OR conception interval*[MeSH Terms]) OR birth interval*[MeSH Terms]) OR birth spacing*[MeSH Terms]) OR inter birth interval*[MeSH Terms]) OR first birth interval*[MeSH Terms]) AND perinatal mortalit*[MeSH Terms]) OR perinatal death*[MeSH Terms]) OR perinatal outcome*[MeSH Terms]) OR adverse perinatal outcome*[MeSH Terms]) OR stillbirth*[MeSH Terms]) OR early neonatal death*[MeSH Terms]) OR early neonatal mortalit*[MeSH Terms]) OR fetal death*[MeSH Terms]) OR pregnancy outcome*[MeSH Terms]) OR adverse pregnancy outcome*[MeSH Terms]) OR birth outcome*[MeSH Terms]) OR adverse birth outcome*[MeSH Terms]) AND Ethiopia).
