# Supplementary material for: Magnitude and trend of perinatal mortality and its relationship with inter-pregnancy interval in Ethiopia: a systematic review and meta-analysis
Source: BMC Pregnancy Childbirth. 2020 Jul 29;20:432. doi: 10.1186/s12884-020-03089-2 (PMC7389567; doi:10.1186/s12884-020-03089-2)
Supplement: Supplementary file 3 — Additional file 3: Table S1. Weaknesses of primary studies and JBI quality scores. [file 12884_2020_3089_MOESM3_ESM.docx]

**Additional file 3: Table S1 Weaknesses of primary studies and JBI quality scores**

| **Study ID** | **Author (year)** | **Weaknesses** | **JBI score** | **Quality status** |
| --- | --- | --- | --- | --- |
| 1 | Sahlemariam Y (1997) | Relay on data of newborns delivered in health institutions; the other alternative of including home deliveries was not considered. Urban newborns only where neonatal mortality was relatively not common as rural | 54.5% | Low risk |
| 2 | EDHS (2000) | No causal relationship of perinatal mortality was established with inter-pregnancy interval or birth interval | 62.5% | Low risk |
| 3 | EDHS (2005) | No causal relationship of perinatal mortality was established with inter-pregnancy interval or birth interval | 62.5% | Low risk |
| 4 | EDHS (2011) | No causal relationship of perinatal mortality was established with inter-pregnancy interval or birth interval. | 62.5% | Low risk |
| 5 | Chekol A  (2011) | Probability sampling techniques not used. Risk factors were not exhaustively included like inter-pregnancy interval. Hence findings were likely to be confounded with other factors too. | 75% | Low risk |
| 6 | Assefa N (2012) | Factors for abortion and stillbirth may not be the same, separate analysis might be needed rather than merging them as pregnancy loss. Only common factors, for both, might be identified here. | 63.6% | Low risk |
| 7 | Wakgari N (2013) | Depends on secondary data, hence it might have incomplete information on confounders. | 87.5% | Low risk |
| 8 | Andargie G (2013) | Confidence interval for some variables remained wide. It might reflect the sample size (1752) was not adequate as the study was community based longitudinal. | 63.6% | Low risk |
| 9 | Worku A (2014) | The study considered small sample size (763) for the cohort study. It could be difficult to establish causal relationship with smaller sample size for relatively rare outcomes in the population. | 90.9% | Low risk |
| 10 | Adane A (2014) | One of the risk factors for stillbirth is inter-pregnancy interval but it was not included in the model. The study lacked precision; it could be due to small sample size (490). | 87.5% | Low risk |
| 11 | Yaya Y (2014) | The study did not consider inter-pregnancy interval or birth interval as risk factor for perinatal outcomes; likely be confounded. Whether maternal mortality resulted in stillbirth or being stillbirth leads to maternal mortality might be difficult to ascertain. It did not stated as limitation | 62.5% | Low risk |
| 12 | Debelew G (2014) | The verbal autopsy was based on mothers’ report of signs and symptoms concerning the underlying causes of neonatal death. This may not be as specific as the clinical diagnosis in identifying the exact cause of death. | 63.6% | Low risk |
| 13 | Abdo R (2016) | Factors of adverse birth outcomes might not be the same so it needs separate analysis for each outcome. Factor like inter-pregnancy interval was not included. | 75% | Low risk |
| 14 | Mengesha H (2016) | As this is a cohort study, it has to define main exposure variable at the beginning. It seems prospective follow up study. Factors were not exhaustively addressed. For example, inter-pregnancy interval is not included. | 63.6% | Low risk |
| 15 | Shifa G (2016) | Cause of mortalities was not ascertained either by using verbal or clinician report even though these have their own limitations. | 50% | Low risk |
| 16 | Berhie K (2016) | It is based on secondary data analysis. Hence it might not address other predictors including inter-pregnancy interval. | 62.5% | Low risk |
| 17 | Asefa D (2016) | Incomplete information due to the nature of secondary data. Factors included might not be adequate. | 50% | Low risk |
| 18 | Aragaw Y (2016) | Factors other than mechanical were not adequately addressed including inter-pregnancy interval | 62.5% | Low risk |
| 19 | Ballard K (2016) | Factors were not exhaustively addressed and adjusted for confounding variables including inter-pregnancy interval | 62.5% | Low risk |
| 20 | Yirgu R (2016) | Category for birth interval was not in line with either WHO recommendation or available in literatures for comparison. Otherwise, it would be good to categorize as <24 and >=24 months rather than <24, 24 and >24 months. Interval falling at exactly 24 month might be rare. | 90% | Low risk |
| 21 | Cherie N (2017) | Factors were not exhaustively addressed and adjusted for confounding variables including inter-pregnancy interval | 75% | Low risk |
| 22 | Dejene T (2017) | Incomplete information due to the nature of secondary data. Factors included might not adequate since it relay on ante partum and intra-partum periods which were more of clinical factors. | 62.5% | Low risk |
| 23 | Mihiretu A (2017) | Incomplete information due to the nature of secondary data. Factors included might not be exhaustive. | 62.5% | Low risk |
| 24 | Lakew D (2017) | Incomplete information due to the nature of secondary data. | 100% | Low risk |
| 25 | Tsegaye B (2018) | Incomplete information due to the nature of secondary data. Factors included might not be exhaustive. No specific report on the number of stillbirths and early neonatal mortalities. | 87.5% | Low risk |
| 26 | Roro E (2018) | Category for birth interval was not in line with either WHO recommendation or available in literatures for comparison. Otherwise, it would be good to categorize as <24 and >=24 months rather than <24, 24 and >24 months. | 90% | Low risk |
| 27 | Goba G (2018) | There were no data on the outcomes of the control group who were discharged before the end of the early neonatal period. It was not determined whether the intra-partum stillbirth occurred during the first or second stage of labor. | 90% | Low risk |
| 28 | Getiye Y (2017) | Incomplete information due to the nature of secondary data. Factors included might not be exhaustive. | 90% | Low risk |
| 29 | Tilahun S (2008) | Category of birth interval was not in a comparable way with other literatures and WHO recommendation. | 90% | Low risk |
| 30 | Tewabe T (2018) | Incomplete information due to the nature of secondary data. Factors were not exhaustively addressed and adjusted for confounding variables including inter-pregnancy interval. | 62.5% | Low risk |
| 31 | Haftu A (2018) | Confounding factors including inter-pregnancy interval were not well addressed to identify independent effects | 81.8% | Low risk |
| 32 | WoldeamanueB (2019) | Category of birth interval was not in line with other literatures. Hence it might be difficult to compare findings.It was dependent on secondary data of five years back. Thus recall bias in reporting perinatal death might affect the results | 87.5% | Low risk |
| 33 | Berhe T (2019) | Relatively small sample size; 570 (wider confidence intervals). | 87.5% | Low risk |
| 34 | Worede D (2019) | Relatively small sample size; 420(wider confidence intervals). Since the study conducted on comprehensive specialized hospital where complicated cases managed it might affect homogeneity of cases and controls as mothers with relatively normal conditions visit lower level health facilities | 90% | Low risk |
| 35 | Gizaw M (2014) | Confounding factors were not exhaustively addressed including inter-pregnancy interval. As it was cohort study main exposure variable was not identified. Information on lost to follow up was not clearly stated. | 45.4 | High risk |
