# Supplementary material for: Magnitude and trend of perinatal mortality and its relationship with inter-pregnancy interval in Ethiopia: a systematic review and meta-analysis
Source: BMC Pregnancy Childbirth. 2020 Jul 29;20:432. doi: 10.1186/s12884-020-03089-2 (PMC7389567; doi:10.1186/s12884-020-03089-2)
Supplement: Supplementary file 4 — Additional file 4: Figure S1. Sensitivity Analysis. [file 12884_2020_3089_MOESM4_ESM.docx]

**Additional file 4 Sensitivity Analysis**

| ****  **PMR (95% CI)**  **Study**  **Figure S1** Forest plot showing pooled perinatal mortality rate (PMR) when each study is removed, Ethiopia.  In figure S1: the horizontal line of each study indicates the 95% confidence interval of the pooled effect size (PMR) when that study is removed. The dot at the middle of the horizontal line indicates the pooled effect size (PMR) when that study is removed. |
| --- |
